# Waste-to-Heat: Thermophysical and Rheological Properties of Fly Ash Dispersions in Deep Eutectic Solvents

**DOI:** 10.3390/molecules31173125

**Published:** 2026-09-07

**Authors:** Bertrand Jóźwiak, Grzegorz Dzido

**Affiliations:** 1 Department of Chemical and Molecular Engineering, Faculty of Process and Environmental Engineering, Lodz University of Technology, Wólczańska 213, 93-005 Łódź, Poland; 2 Department of Chemical Engineering and Process Design, Faculty of Chemistry, Silesian University of Technology, Strzody 7, 44-100 Gliwice, Poland; grzegorz.dzido@polsl.pl

**Keywords:** deep eutectic solvents, fly ash, heat transfer fluids, waste valorization, thermal conductivity, rheology, stability

## Abstract

Deep eutectic solvents (DESs) have emerged as promising heat-transfer fluids. Incorporating near-zero-cost waste-derived particles into DESs may enhance their thermophysical performance while supporting circular economy principles. To the best of our knowledge, this is the first report on the use of municipal solid waste incineration fly ash (FA) in DES-based heat-transfer fluids. This study investigates the thermophysical, rheological, morphological, and stability properties of dispersions prepared using choline chloride/ethylene glycol (ChCl/EG) DESs with molar ratios of 1:5 and 1:10. Dispersions containing 2–20 wt% FA were prepared by ultrasonication without particle milling or surfactants and characterized using SEM–EDS, laser diffraction, multiple light scattering, transient hot-wire method, and rotational rheometry at 25 °C. Increasing FA loading resulted in a linear increase in thermal conductivity, reaching maximum enhancements of 19% for ChCl/EG (1:10) and 13% for ChCl/EG (1:5) at 20 wt%. Particle size analysis indicated effective deagglomeration after ultrasonication at low FA concentrations (2–5 wt%), whereas higher loadings promoted gradual re-agglomeration. All dispersions exhibited Newtonian liquid-like behavior, with viscosity increasing with FA concentration while remaining below 90 mPa·s. During the initial 3 h observation period, increasing FA loading reduced dispersion stability, whereas a higher ChCl content improved stability. Overall, the results demonstrate the potential of waste-derived FA in DES-based heat-transfer fluids and support its use within the waste-to-heat concept, linking waste valorization with heat-transfer applications.

## 1. Introduction

The continuous growth of the global population and rapid industrialization have led to a substantial increase in energy demand, placing significant pressure on energy conversion and management systems. Consequently, improving the efficiency of heat transfer has become a key research priority. Conventional heat-transfer fluids, such as water, ethylene glycol (EG), and various oils, have traditionally played a central role in thermal systems. However, these fluids often reach their functional limits due to inherently low thermal conductivity, which hinders the efficiency of heat exchangers, solar collectors, and automotive cooling systems [1,2,3]. To address these constraints, researchers have increasingly investigated particle-enhanced heat-transfer fluids ranging from micron-sized dispersions to nanofluids. Although nanofluids, introduced by Choi in 1995 [4], have received the greatest attention, micron-sized dispersions remain an attractive alternative owing to their lower cost and reduced processing requirements.

In the transition towards a circular economy, industrial by-products are increasingly being re-evaluated as sources of functional materials [5,6,7]. Fly ash (FA) is one of the most abundant residues generated during coal combustion and municipal solid waste incineration, with millions of tonnes produced worldwide each year. Despite its significant resource potential, a substantial fraction is still disposed of in landfills or temporary storage facilities, posing long-term environmental and economic challenges. Chemically, FA is a complex and heterogeneous material composed of multiple inorganic phases. Depending on its origin, it may contain varying proportions of metal and metalloid oxides, salts, and residual carbon, making it an abundant, readily available, and cost-effective alternative to commercial solid additives used in heat-transfer fluids [8,9].

Dispersions prepared directly from untreated micron-sized FA have received little attention in the literature. To date, only a few studies have investigated the preparation and thermophysical properties of water-based FA nanofluids. A two-step method is commonly employed, in which particles are dispersed in a base fluid and subsequently subjected to intensive mechanical stirring and ultrasonication to ensure a homogeneous suspension [10,11,12]. Additionally, high-energy ball milling is frequently employed to substantially reduce the particle size of FA, increasing its specific surface area and enhancing its reactivity and dispersibility [2,10]. Studies have demonstrated that adding FA to distilled water improves thermal conductivity by approximately 12% and 32% at 0.5 vol% and 4.0 vol% of particles at 60 °C, respectively [10,12]. Hybrid nanofluids, which combine two or more different solid materials in a single base fluid, can provide even greater enhancements owing to synergistic interactions between the particles. For instance, a water-based hybrid FA/Cu (4:1) nanofluid exhibited a 50% increase in thermal conductivity at 4.0 vol% particle loading and 60 °C [12]. Likewise, water-based hybrid FA/GO nanofluids achieved thermal conductivity increases of 45.6% and 41.3% for FA/GO ratios of 1:1 and 7:3, respectively, at a particle concentration of 1.0 vol% at 60 °C [2]. These improvements are generally attributed to mechanisms such as Brownian motion, particle clustering, and micro-convection, which collectively facilitate heat transport within the suspension [2].

Water-based FA dispersions typically exhibit Newtonian behavior, with dynamic viscosity increasing with particle concentration. For example, at a particle loading of 0.5 vol% and 30 °C, the viscosity increased by approximately 13% compared with the pure base fluid [10]. Interestingly, the incorporation of FA into water-based GO dispersions restored Newtonian behavior over the investigated shear rate range of 10–250 s^−1^ [2]. Neat GO nanofluids showed shear-thinning behavior, whereas hybrid FA/GO (1:1 and 7:3) nanofluids exhibited nearly constant viscosity. This effect has been attributed to the spherical morphology of FA, which facilitates smoother flow and reduces viscous resistance compared with flake-like or irregularly shaped particles [2].

The practical utility of FA-based dispersions has been demonstrated in several thermal engineering applications. In the field of solar energy, studies on flat-plate solar collectors using zigzag-shaped tubes have shown that hybrid water-based FA/Cu (4:1) nanofluids at concentrations from 0.01 to 1.5 vol% significantly enhance the Nusselt number and convective heat-transfer coefficient by approximately 33% and 41% compared with the base fluid, respectively [13]. In automotive and electromobility sectors, water/EG (3:2)-based FA nanofluids with a particle concentration of 2 vol% have proven effective for heavy-vehicle radiators [14]. At a Reynolds number of 8000, the overall heat-transfer coefficient increased by 21% relative to the corresponding base fluid. Most recently, FA nanofluids have emerged as promising coolants for electric vehicle battery thermal management systems, where they help maintain lithium-ion batteries within the optimal operating temperature range of 15–35 °C, thereby reducing the risk of thermal runaway [15,16]. In these systems, FA nanoparticles are typically dispersed in water or water/EG (7:3) mixtures at concentrations ranging from 0.1 to 5 vol%, enhancing the thermal conductivity of the base fluid and improving heat transfer while maintaining acceptable flow characteristics.

Despite the growing body of work on FA dispersed in conventional (molecular) liquids, there is a significant research gap regarding its use in green designer solvents. Deep eutectic solvents (DESs) are particularly attractive as base fluids for particle-based heat-transfer systems because they combine several features that are difficult to obtain simultaneously in conventional molecular liquids. Their very low vapor pressure can reduce evaporation losses and emissions during operation, while their relatively high thermal stability allows their use over a broader temperature range [17,18]. In addition, DES properties can be tailored by changing the chemical nature and molar ratio of their components, providing a convenient means of adjusting viscosity, density, polarity, thermal conductivity, and other physicochemical properties to the requirements of a specific heat-transfer application [19]. From a technological perspective, DESs can be prepared by simple mixing of their components, generally without the need for complex synthesis, purification, or solvent removal, thereby reducing process complexity and potentially lowering energy consumption and waste generation [20]. Their ionic and strongly hydrogen-bonded structure may also promote favorable interactions with dispersed solid particles, potentially facilitating the formulation of stable dispersions [19]. Among DESs, systems based on choline chloride (ChCl) and EG are particularly attractive for heat-transfer applications due to their favorable thermal conductivity, cost-effectiveness, relatively low viscosity, and low volatility [21,22].

To the best of our knowledge, no previous study has systematically investigated DES-based dispersions containing either micron-sized or nanosized FA for heat-transfer applications. Building on our previous work [22], which demonstrated that replacing expensive carbon nanomaterials with low-cost oxide particles improved the economic viability of DES-based nanofluids, the present study employs municipal solid waste incineration FA as the dispersed phase. The use of this abundant waste-derived material further enhances the economic and environmental rationale of the proposed system by reducing material costs, promoting waste valorization, and supporting circular economy principles.

This study investigates the influence of FA concentration and DES composition on the thermal conductivity, dynamic viscosity, particle size distribution, and initial time-dependent stability of ChCl/EG-based dispersions. The results provide new insights into the behavior of DES-based dispersions prepared from micron-sized, waste-derived FA and support the development of cost-effective heat-transfer fluids within the waste-to-heat concept.

## 2. Results and Discussion

### 2.1. FA Characterization

**Figure 1:**
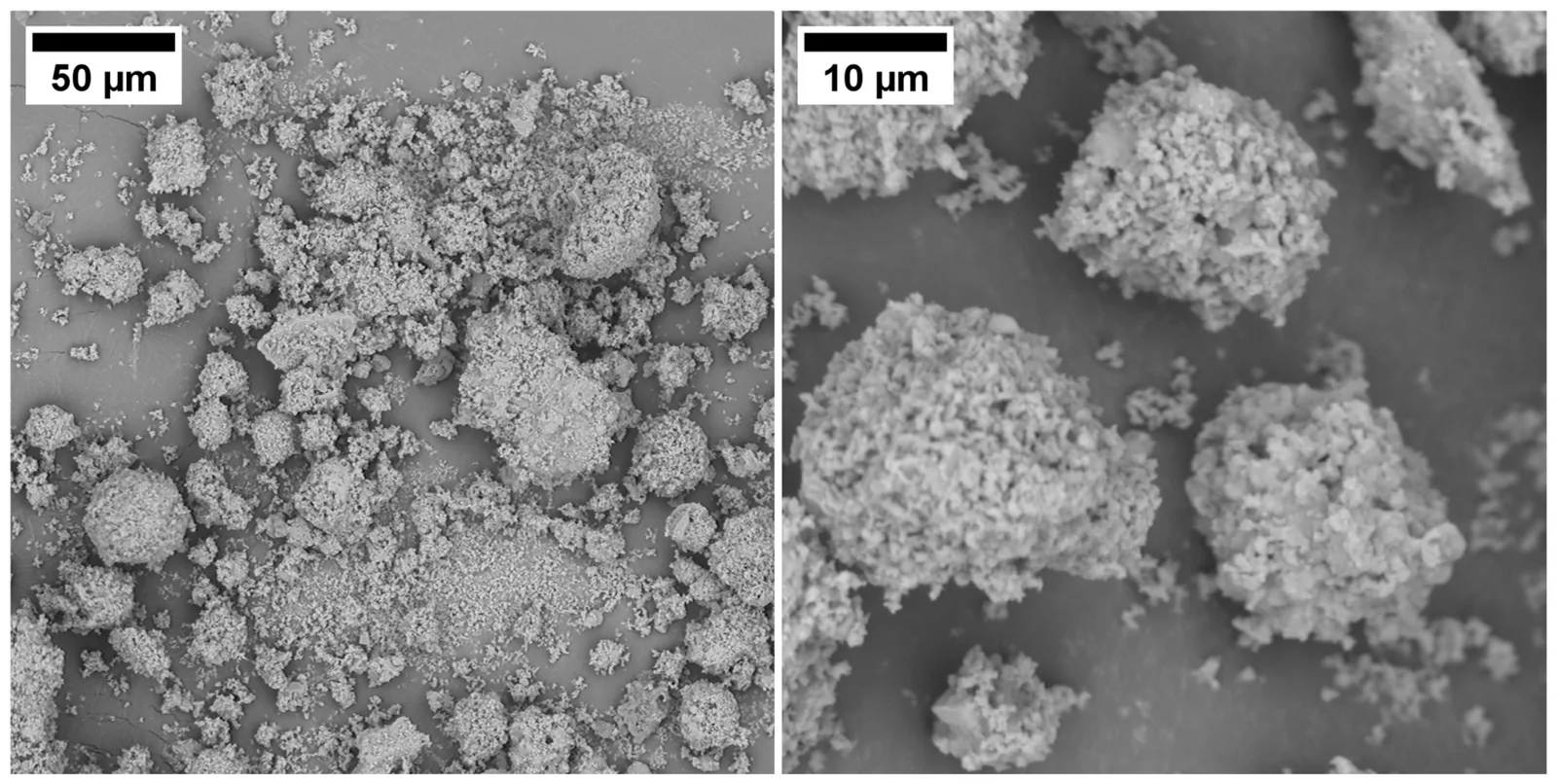
SEM micrograph of raw FA: 1000× (**left**) and 5000× (**right**).

Figure 1 presents scanning electron microscopy (SEM) micrographs of the raw FA. The material consists predominantly of irregular, highly agglomerated particles with rough and porous surfaces. Rather than isolated primary particles, FA forms compact aggregates composed of numerous finer fragments, indicating pronounced interparticle adhesion. Such morphology results from the complex sequence of melting, condensation, and solidification processes occurring during waste combustion and is consistent with previous observations of municipal solid waste incineration FA [23]. The rough surface texture and porous structure may increase the specific surface area, thereby promoting particle–liquid interactions upon dispersion.

The average elemental composition of FA is shown in Table 1. The energy-dispersive X-ray spectroscopy (EDS) analysis showed relatively high signals for carbon, oxygen, calcium, and chlorine. The calcium- and chlorine-rich composition is consistent with values commonly reported for FA derived from municipal solid waste incineration [24]. The carbon signal, however, requires cautious interpretation because EDS provides localized surface information rather than a bulk elemental composition. Independent characterization of FA from the same municipal waste incineration facility reported total organic carbon contents of 5.80–8.15 wt%, indicating that the 26.33 wt% measured by EDS should not be interpreted as the bulk carbon content. This distinction is particularly important when comparing the present material with other carbon-rich solid wastes, including coal-based residues, for which the distribution and association of carbon with mineral phases can vary substantially [25,26].

**Figure 2:**
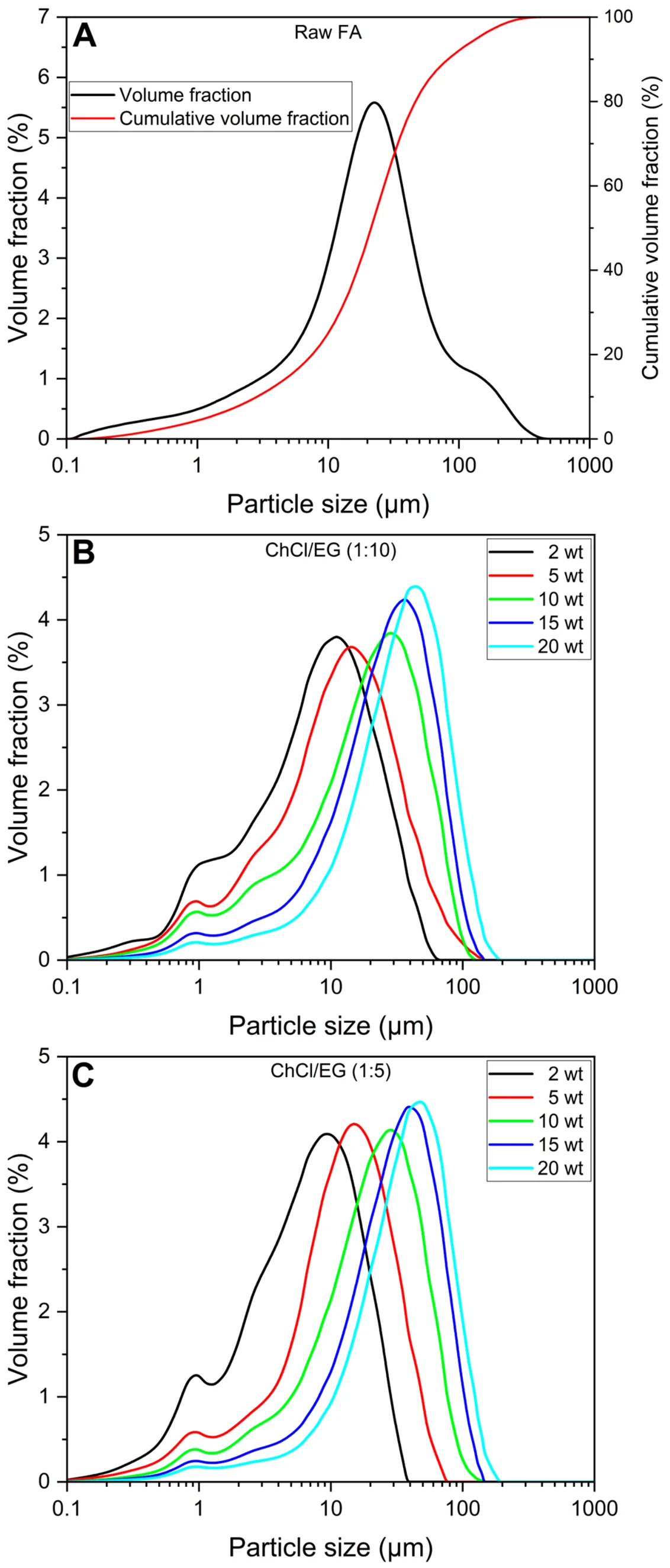
Volume-based particle size distribution of (**A**) raw FA, (**B**) FA dispersed in ChCl/EG (1:10), and (**C**) FA dispersed in ChCl/EG (1:5).

The particle size distributions of raw FA and FA dispersed in DESs are presented in Figure 2, while the corresponding characteristic diameters are summarized in Table 2. The raw FA exhibited a broad distribution (*Dv*(50) = 21.3 μm, span = 3.74), indicating a highly polydisperse material composed of fine particles and coarse agglomerates. Kumar et al. [27] reported a similar median particle diameter (*Dv*(50) = 26.6 μm) for coal FA, despite the different origin of the material. In the present study, at low FA concentrations (2–5 wt%), ultrasonication markedly reduced all measured particle diameters compared with the raw FA, indicating efficient deagglomeration. A further increase in FA loading progressively increased the diameters, suggesting gradual re-agglomeration driven by enhanced particle–particle interactions. These may involve van der Waals and electrostatic forces, while capillary effects within the rough and porous FA structure may also contribute. The polar and hydrogen-bonding character of ChCl/EG may facilitate wetting of the FA surface. However, the relative strength of these interactions and wettability were not directly measured and therefore remain plausible mechanisms. The differences between the two DES compositions were most apparent at the lowest FA concentrations, where smaller characteristic diameters of FA were observed for ChCl/EG (1:5). This may be associated with the higher viscosity of the continuous phase and stronger interactions between the particle surface and the ChCl-rich medium, thereby suppressing re-agglomeration. In addition, the reduction in the span parameter from 3.74 for the raw FA to 2.04–2.73 for the dispersions indicates that ultrasonication resulted in a narrower size distribution with reduced polydispersity.

### 2.2. Dispersion Stability

**Figure 3:**
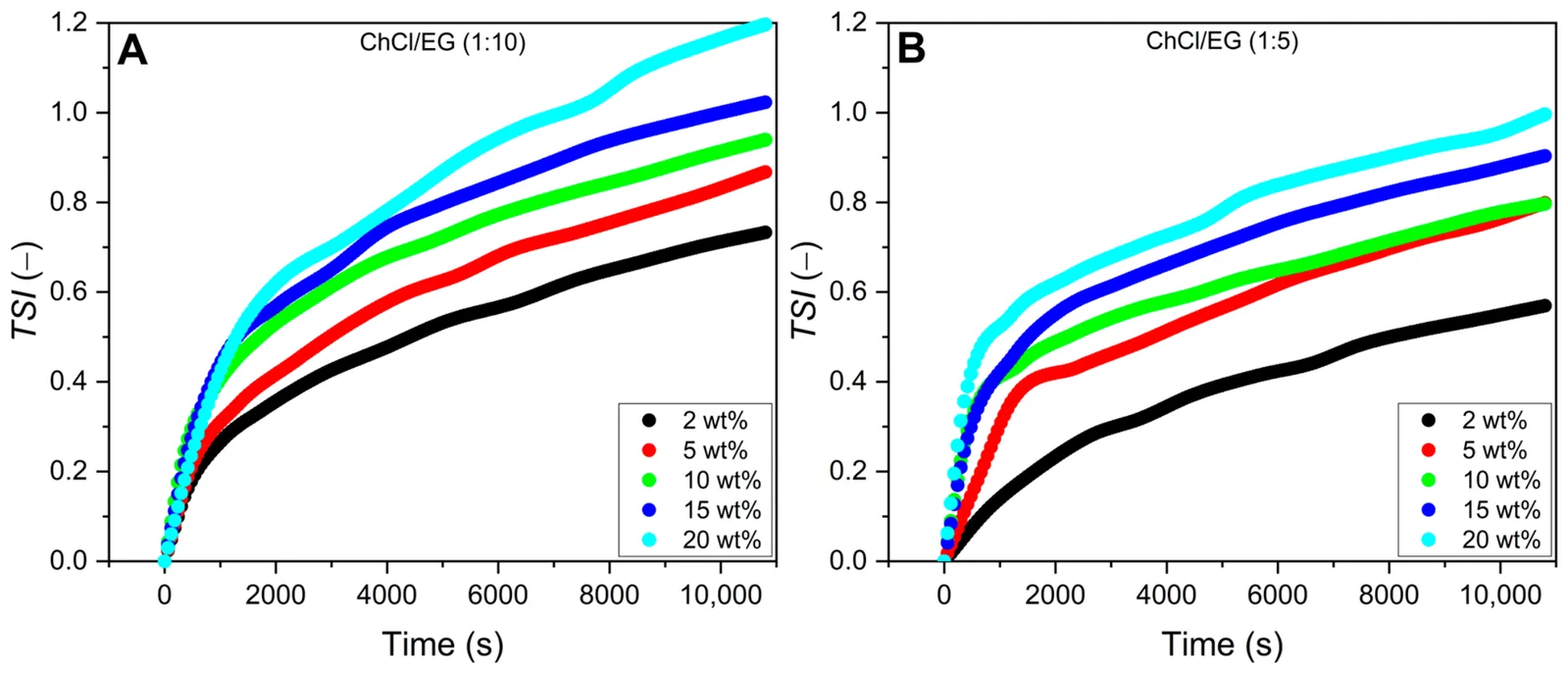
*TSI* as a function of time for DES-based FA dispersions at 25 °C: (**A**) ChCl/EG (1:10) and (**B**) ChCl/EG (1:5).

The initial time-dependent stability behavior of the DES-based FA dispersions was evaluated using the turbiscan stability index (*TSI*), with the results presented in Figure 3. The measurements were conducted over a 3 h observation window and are therefore intended to characterize the initial or ultra-short-term destabilization behavior, and should not be interpreted as evidence of long-term storage or operational stability. For all investigated systems, the *TSI* increased with time and with FA loading, indicating progressive destabilization. A similar concentration-dependent reduction in dispersion stability has been reported for water-based FA nanofluids, where the absolute zeta potential decreased from 37.5 mV at 0.5 vol% to 31.2 mV at 4.0 vol% FA, indicating progressively stronger particle interactions [12].

A clear influence of the DES composition was also observed. At comparable FA concentrations, dispersions prepared with ChCl/EG (1:5) generally exhibited lower *TSI* values than those based on ChCl/EG (1:10), demonstrating improved stability, in agreement with previous studies on DES-based dispersions [28,29]. This may be attributed to the higher viscosity and more extensive hydrogen-bonding network associated with the higher ChCl content, which can reduce particle mobility, thereby delaying destabilization.

To further elucidate the destabilization mechanism, the time-resolved backscattering (BS) profiles recorded during the measurements were examined for representative low- and high-loading dispersions (2 and 20 wt% FA) in both DES systems (Figures S1–S4 in Supplementary Materials). The BS profiles showed a progressive decrease in the signal in the upper part of the measurement cell, while the lower part exhibited a corresponding increase or accumulation of the signal. This spatial redistribution of the BS is consistent with particle migration toward the bottom of the cell and indicates gravitational sedimentation as the predominant observed destabilization mechanism. The changes were more pronounced at 20 wt% FA than at 2 wt% FA, consistent with the higher *TSI* values observed at elevated particle loadings. In contrast, the relatively small changes across the central region of the measurement cell do not provide clear evidence of a homogeneous destabilization process such as bulk flocculation. Re-agglomeration may nevertheless contribute to the formation of larger particle structures and thereby facilitate their gravitational settling. The lower extent of BS signal changes observed for the ChCl/EG (1:5) dispersions is consistent with their lower *TSI* values and indicates slower particle migration under otherwise comparable conditions.

### 2.3. Thermal Conductivity

**Figure 4:**
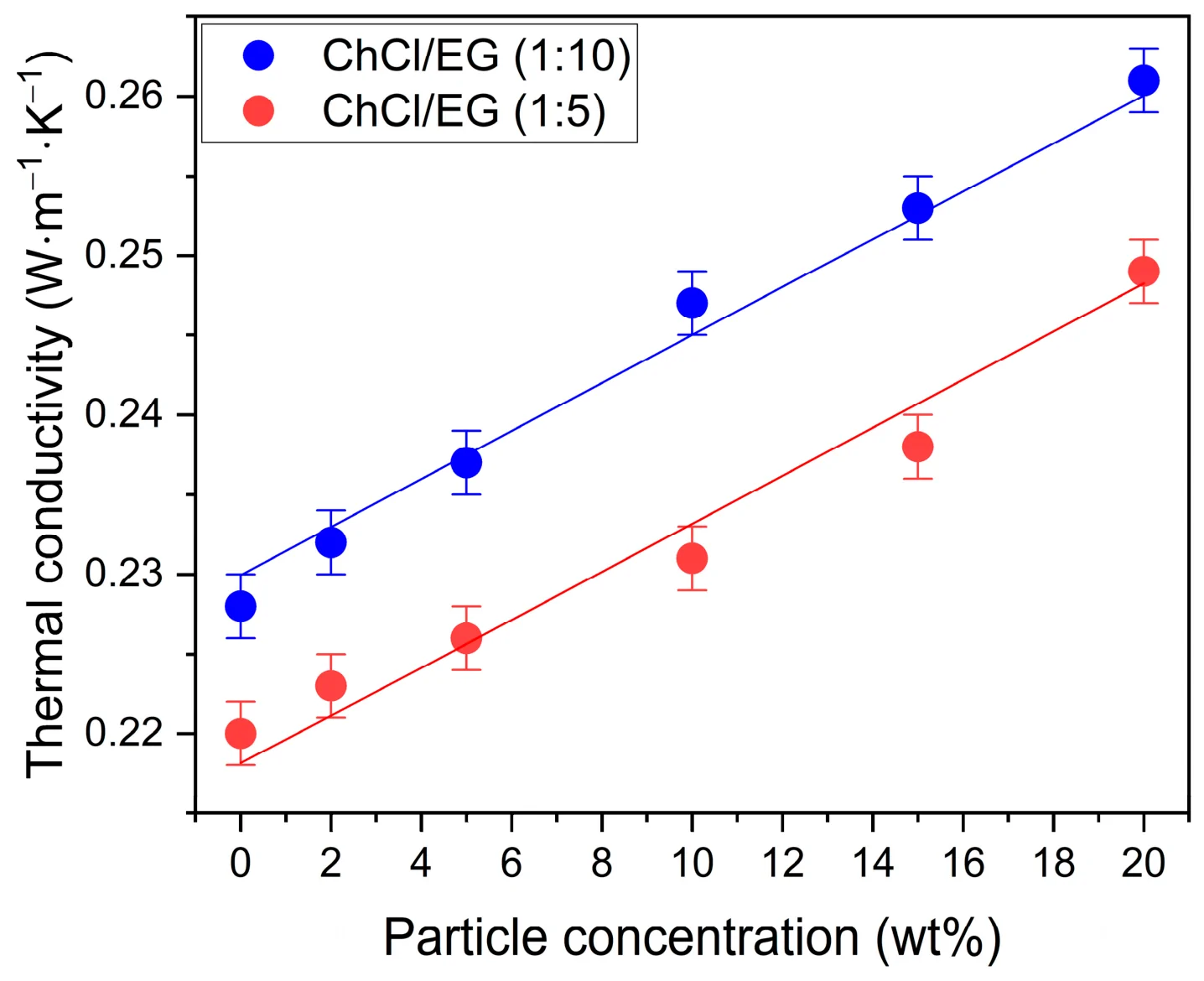
Thermal conductivity of ChCl/EG-based FA dispersions at 25 °C. Solid lines represent the predictions of the empirical model expressed by Equation (1).

The thermal conductivity of the FA dispersions as a function of particle concentration is presented in Figure 4. For both DES systems, thermal conductivity increased almost linearly with FA concentration, indicating that the incorporation of the heterogeneous solid phase progressively modified heat transport within the DES. Depending on its composition, density, moisture content, and porosity, the effective thermal conductivity of bulk FA reported in the literature typically ranges from 0.6 to 1.7 W·m^–1^·K^–1^ [11,30], compared with approximately 0.22–0.23 W·m^–1^·K^–1^ for the investigated DESs. These literature values should not, however, be interpreted as the intrinsic thermal conductivity of the specific FA used in this study. The carbonaceous fraction may also contribute to heat transport within the dispersed phase, although its individual contribution cannot be quantified from the present measurements.

The dispersed particles provide additional heat-conduction pathways, while decreasing interparticle distances at higher FA loadings may facilitate heat transfer through particle–particle interactions and transient particle-mediated pathways, thereby reducing the overall thermal resistance of the dispersion. In addition, heat transport at the solid–liquid interface may be influenced by the specific molecular environment provided by the DES. The ChCl/EG phase is characterized by an extensive hydrogen-bonding network, while the ionic nature of ChCl may promote interactions with polar and ionic surface sites present in the heterogeneous FA. These interactions may affect the local organization and mobility of DES molecules near the particle surface and thereby influence heat transport across the solid–liquid interface. The magnitude of this contribution is expected to depend on the ChCl/EG composition, which modifies both the strength of the hydrogen-bonding network and the viscosity of the continuous phase [31].

The rough and porous morphology of the FA observed by SEM may also influence thermal transport by modifying the effective particle–liquid contact area. Previous molecular dynamics studies have shown that surface roughness can affect thermal conductance at solid–liquid interfaces, with the magnitude of this effect depending on the surface topography and the strength of interfacial interactions [32]. However, surface roughness was not quantitatively characterized in the present study; therefore, its contribution to the observed thermal conductivity enhancement should be regarded as a possible rather than demonstrated mechanism.

To further quantify the dependence of thermal conductivity on FA loading and DES composition, the experimental data were fitted using an empirical equation:

(1) λ=0.24179+0.0015050x−0.11833r

where *λ* is the dispersion thermal conductivity (W·m^–1^·K^–1^), *x* is the FA loading (wt%), and *r* is the ChCl/EG molar ratio, defined as 0.1 for 1:10 and 0.2 for 1:5. The model showed a very good agreement with the experimental data, with R^2^ = 0.984 and RMSE = 0.00158 W·m^–1^·K^–1^ (Figure 4).

The maximum enhancement in thermal conductivity reached approximately 19% (0.261 W·m^–1^·K^–1^) for ChCl/EG (1:10) and 13% (0.249 W·m^–1^·K^–1^) for ChCl/EG (1:5) at a FA loading of 20 wt% at 25 °C. In comparison, Kanti et al. [12] reported a thermal conductivity increase of approximately 31% for water-based FA nanofluids containing 4 vol% particles (corresponding to approximately 10 wt% in the present study) at 30 °C. Although the enhancement observed for the DES-based dispersions was lower, this difference can be attributed to the intrinsically higher viscosity and stronger hydrogen-bonding network of DESs, as well as to the considerably smaller particle size (ca. 14.5 nm) employed by Kanti et al. [12], obtained through an additional 60-h high-energy milling process. The much larger median particle diameter (*Dv*(50) = 6.0–36 µm) of the FA used in the present study is associated with a lower specific surface area and reduced particle–fluid interfacial interactions, thereby limiting the enhancement in thermal conductivity. Nevertheless, the observed increase in thermal conductivity of up to 19% demonstrates that micron-sized FA provides a meaningful improvement in the performance of DES-based heat-transfer fluids while offering a considerably simpler, faster, and more cost-effective preparation procedure.

Throughout the investigated FA concentration range, the ChCl/EG (1:10) dispersions exhibited, on average, approximately 5.0% higher thermal conductivity than the corresponding ChCl/EG (1:5) dispersions. Increasing the ChCl content in the DES increases the viscosity of the solvent, which restricts molecular mobility and reduces heat transport efficiency. Similar observations have also been reported in previous studies on DESs and DES-based nanofluids [21,29,33]. For instance, Jafari et al. [29] reported that increasing the ChCl/EG molar ratio from 1:5 to 1:2 reduced the thermal conductivity of DES-based MgO nanofluids over the investigated particle loadings (1–10 wt%) by an average of 5.6%.

### 2.4. Rheological Properties

**Figure 5:**
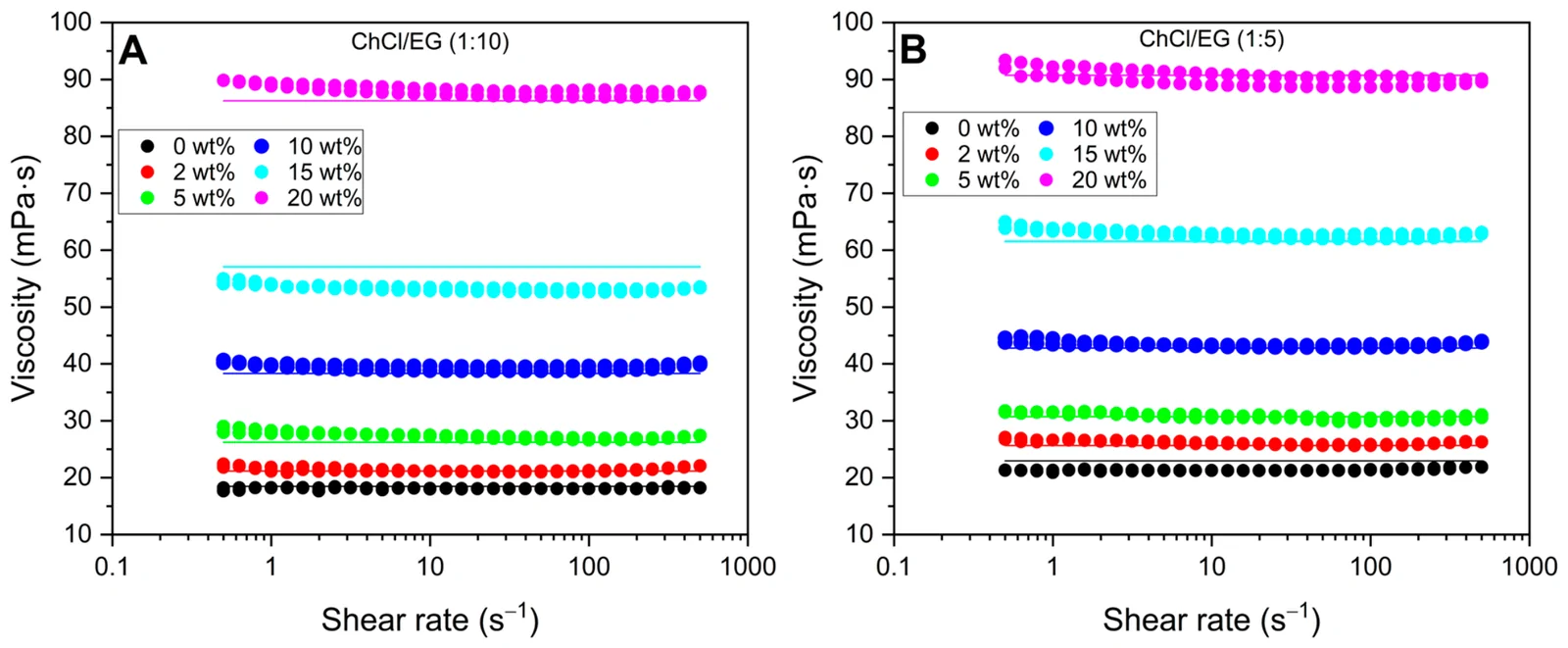
Viscosity curves as a function of shear rate for DES-based FA dispersions at 25 °C: (**A**) ChCl/EG (1:10) and (**B**) ChCl/EG (1:5). Solid lines represent the predictions of the empirical model expressed by Equation (2).

The rheology of the DES-based FA dispersions is presented in Figure 5. All investigated samples exhibited Newtonian behavior, as evidenced by the nearly constant viscosity throughout the investigated shear rate range of 0.5–500 s^–1^. The results are consistent with those reported by Kumar et al. [27,34], who found that water-based FA slurries remained Newtonian within the range of 50–300 s^–1^ up to 30 wt% solids, whereas higher particle loadings resulted in Bingham plastic behavior accompanied by a progressive increase in yield stress. Similarly, Kanti and Maiya [2] demonstrated that the incorporation of FA into water-based GO dispersions (FA/GO ratios of 1:1 and 7:3, total particle loading 0.75–1 vol%) restored Newtonian behavior over the shear rate range of 10–250 s^–1^. In addition, the ascending and descending viscosity curves obtained in the present study overlapped, indicating the absence of thixotropy. These findings suggest that no significant time-dependent microstructural changes occurred during shear and that the dispersions remained stable under the applied flow conditions.

To further analyze the rheological behavior, the dependence of viscosity on FA loading and DES composition was described using an empirical model:

(2) $$\mu =13.993e^{0.088270x}+44.746r$$

where *μ* is the dispersion viscosity (mPa·s), *x* is the FA loading (wt%), and *r* is the ChCl/EG molar ratio (0.1 for 1:10 and 0.2 for 1:5). The model provided an excellent fit to the experimental data with R^2^ = 0.996 and RMSE = 1.45 mPa·s (Figure 5).

For both DES systems, the viscosity increased monotonically with FA concentration (Table 3), reaching maximum values of 87.8 mPa·s for ChCl/EG (1:10) and 89.9 mPa·s for ChCl/EG (1:5) at 20 wt% FA. This behavior is primarily attributed to the increasing particle loading, which restricts the mobility of the liquid phase and enhances hydrodynamic interactions between suspended particles. At higher particle loadings, the average interparticle distance decreases, resulting in more frequent particle–particle interactions and, consequently, greater resistance to flow. At 25 °C and FA loading of 10 wt%, the viscosity of the ChCl/EG (1:5) dispersion reached 43.4 mPa·s, which is comparable to that previously reported for Al_2_O_3_ (45.8 mPa·s), lower than that of SiO_2_ (56.1 mPa·s), and higher than those of TiO_2_ (38.3 mPa·s) and CuO (28.5 mPa·s) nanofluids prepared in the same DES [22]. Unlike the previously investigated DES-based metal and metalloid oxide nanofluids, which exhibited shear-thinning behavior within the shear rate range of 12.9–258 s^–1^ at particle loadings of 15 wt% and above [22], all DES-based FA dispersions remained Newtonian even at 20 wt%, indicating that the critical concentration required for the formation of a continuous particle network was not reached [35].

Throughout the investigated FA concentration range, the ChCl/EG (1:5) dispersions exhibited consistently higher viscosities than the corresponding ChCl/EG (1:10) systems, with the difference varying from 2.4% to 22.0%, depending on the particle loading (Table 3). This behavior may be attributed to the stronger hydrogen-bonding network associated with the higher ChCl content, which reduces molecular mobility, while the addition of FA further amplifies this effect through increased hydrodynamic resistance [28,36].

Even at the highest FA loading, all investigated DES-based dispersions remained liquid-like and Newtonian, with viscosities below 90 mPa·s at 25 °C. Such moderate viscosity, combined with the enhanced thermal conductivity, is advantageous for heat-transfer applications because it limits pumping power requirements while improving overall thermal performance. Furthermore, the absence of thixotropy indicates that the rheological properties remain stable during continuous circulation, which is beneficial for practical operation in closed-loop systems. Together with the use of a waste-derived solid phase, these characteristics support the development of cost-effective DES-based heat-transfer fluids within the waste-to-heat concept.

## 3. Materials and Methods

### 3.1. Materials

The dispersed phase consisted of FA obtained from the Ekospalarnia Kraków municipal waste incineration plant (Kraków, Poland). The material was collected from the flue-gas treatment system. The plant primarily treats mixed municipal refuse, including fractions originating from mechanical processing, as well as bulky and renovation materials. The incineration process is carried out in a grate-fired system, with temperatures reaching approximately 1000 °C in the upper combustion chamber, while post-combustion is maintained above 850 °C with a flue-gas residence time exceeding 2 s.

The material was sieved through a 250 µm mesh to remove coarse particles and agglomerates, yielding a more homogeneous sample for subsequent physicochemical characterization. After sieving, the FA was used directly for dispersion preparation without any additional grinding or milling.

The base fluids were low-viscosity DESs (<22 mPa·s at 25 °C) composed of ChCl (Pol-Aura, Zabrze, Poland) and EG (Avantor Performance Materials, Gliwice, Poland) at molar ratios of 1:5 and 1:10. The 1:5 ratio was selected based on its established use in previous studies [21,22,29,37], while the 1:10 ratio was chosen to obtain a lower-viscosity DES for comparison. ChCl acted as the hydrogen bond acceptor and EG as the hydrogen bond donor. The composition, purity, and preparation procedure of the DESs have been described in detail elsewhere [21].

### 3.2. Sample Preparation

The dispersions were prepared by incorporating 2, 5, 10, 15, and 20 wt% of FA into the base DESs and processing the mixtures with an ultrasonic homogenizer VCX 750 equipped with a standard CV33 probe (Sonics & Materials, Newtown, CT, USA). Sonication was carried out in continuous mode for 10 min at 20 kHz, 75 W, and 70% amplitude. Throughout treatment, the samples were kept in a water-jacketed vessel to dissipate heat and prevent moisture uptake. No additional dispersants, surfactants, or stabilizing agents were added.

### 3.3. Morphological and Elemental Characterization

The morphology of the raw FA was analyzed by SEM using a Phenom ProX instrument (Thermo Fisher Scientific, Waltham, MA, USA) operated at an accelerating voltage of 10 kV. Prior to imaging, the material was mounted on aluminum stubs with conductive double-sided tape and coated with a 2 nm gold layer using a Q150R ES vacuum sputter coater (Quorum Technologies, Laughton, UK). Micrographs were acquired at magnifications of 1000× and 5000×. The local elemental composition of the FA was determined by SEM coupled with EDS using a silicon drift detector. Point analyses were performed at multiple locations, and the results were reported as average weight percentages.

### 3.4. Particle Size Analysis

The particle size distribution was determined by laser diffraction using a PSA 1190 analyzer (Anton Paar, Graz, Austria) equipped with three solid-state laser diodes operating at 830 nm (1.5 mW and 2 mW) and 635 nm (5 mW). Raw FA was measured in dry mode, where compressed air was used to promote deagglomeration before optical detection, while the dispersions were analyzed in wet mode with water as the dispersing medium directly in the measurement cell. For wet measurements, the sample was gradually added dropwise into the dispersant under continuous mechanical stirring until the desired obscuration level (approximately 5%) was reached. Data were evaluated using the Mie scattering model. The refractive and absorption indices of FA particles were set to *n* = 1.55 and *k* = 0.10, respectively. The refractive index of the continuous medium was set to *n* = 1.33 for water and *n* = 1.00 for air. Results were reported as volume-based particle size distributions. Characteristic diameters *Dv*(10), *Dv*(50), and *Dv*(90), corresponding to the 10th, 50th (median), and 90th percentiles of the cumulative volume curve, were determined. In addition, the De Brouckere mean diameter *D*[4,3] was calculated. The width of the distribution was expressed using the span, defined as [*Dv*(90) − *Dv*(10)]/*Dv*(50). Each value represents the average of at least three consecutive measurements. The repeatability of the measurements, expressed as the relative standard deviation (RSD), was 5%.

### 3.5. Stability Analysis

The initial time-dependent stability of the dispersions, reflecting their resistance to destabilization processes, was evaluated using a Turbiscan Lab Expert (Formulaction, Toulouse, France) based on the multiple light scattering technique. Immediately after preparation, each dispersion was transferred into a cylindrical glass cell (20 mm diameter) and analyzed without delay. The instrument used an infrared light source (880 nm) and two optical detectors positioned at 180° (transmission) and 45° (BS) relative to the incident beam. Owing to the opaque nature of the dispersions, the stability analysis was based on the BS signal recorded over the entire sample height with a spatial resolution of 40 μm. Measurements were performed at 25 °C for 3 h, with scans acquired every 1 min. Stability was quantified using the *TSI*, which was calculated by the instrument software TurbiSoft 2.1.0.52 from temporal variations in the BS signal according to equation:

(3) $$TSI=\sum_{i=1}^{m}\frac{\left|{BS}_{i}\left(h\right)-{BS}_{i-1}\left(h\right)\right|}{H}$$

where *H* is the total sample height (from the bottom of the cell to the meniscus), *h* is the vertical measurement position within the sample, *i* is the scan number, *m* is the total number of scans, and ${BS}_{i}\left(h\right)$ is the BS signal measured at position *h* during the *i*-th scan. Lower *TSI* values correspond to smaller changes in the BS signal and, consequently, greater dispersion stability, whereas increasing *TSI* reflects progressive destabilization. Further details of the *TSI* calculation are provided elsewhere [38].

### 3.6. Thermal Conductivity Measurements

The thermal conductivity of the dispersions was measured at 25 °C using a KD2 Pro Thermal Properties Analyzer equipped with a KS-1 sensor (Decagon Devices, Pullman, WA, USA), based on the transient hot-wire technique [39]. Prior to analysis, the samples were thermostated in a jacketed beaker for 15 min to ensure thermal equilibrium. This was verified using the KD2 Pro temperature sensor, which indicated a change of no more than 0.01 °C after 15 min. All measurements were carried out in triplicate, and the reported values represent the averages. The estimated measurement uncertainty was ±0.002 W·m^–1^·K^–1^. A detailed description of the experimental procedure has been provided elsewhere [21].

### 3.7. Rheological Measurements

The viscosity of the dispersions was determined at 25 °C using a Physica MCR 301 rotary rheometer (Anton Paar, Graz, Austria) equipped with a CC27 concentric cylinder geometry. Prior to testing, the samples were thermostated for 15 min to ensure thermal equilibrium. The measurements were performed in three consecutive steps: (i) a shear rate sweep from 0.5 s^–1^ to 500 s^–1^ (10 points per logarithmic decade, without time settings), (ii) a constant shear rate of 500 s^–1^ maintained for 60 s, and (iii) a reverse shear rate sweep from 500 s^–1^ to 0.5 s^–1^ (10 points per logarithmic decade, without time settings). For samples exhibiting Newtonian behavior, the viscosity was calculated as the arithmetic mean over the entire investigated shear rate range. The repeatability of the viscosity measurements was 2.5% (RSD).

## 4. Conclusions

To the best of our knowledge, this study provides the first report on the use of municipal solid waste incineration FA in DESs for heat-transfer applications. The results demonstrate that micron-sized FA can be successfully utilized as a low-cost dispersed phase in ChCl/EG-based DESs, providing an alternative to conventional oxide fillers and supporting the waste-to-heat concept. The main conclusions are as follows:

- The incorporation of FA resulted in a linear increase in the thermal conductivity of both DES systems, reaching maximum enhancements of 19% (0.261 W·m^–1^·K^–1^) for ChCl/EG (1:10) and 13% (0.249 W·m^–1^·K^–1^) for ChCl/EG (1:5) at 20 wt% and 25 °C. The lower-viscosity ChCl/EG (1:10) system consistently exhibited higher thermal conductivity.
- All investigated dispersions maintained Newtonian behavior throughout the studied concentration range (up to 20 wt%) and showed no thixotropic effects. Although viscosity increased with FA loading, all systems remained below 90 mPa·s at 25 °C.
- Despite the absence of stabilizing agents, the dispersions showed relatively good resistance to initial destabilization over the 3 h observation period, although the extent of changes increased with FA loading. The ChCl/EG (1:5) dispersions exhibited lower *TSI* values, indicating slower initial destabilization than the corresponding ChCl/EG (1:10) systems, which may be related to their higher viscosity and differences in the hydrogen-bonding network.
- The combination of enhanced thermal conductivity, moderate viscosity, relatively good initial stability, simple surfactant-free preparation, and the use of an abundant waste-derived material highlights the potential of DES-based FA dispersions as cost-effective heat-transfer fluids.

Future work should focus on evaluating the long-term stability, specific heat capacity, pumping power requirements, convective heat-transfer performance, and environmental safety of these dispersions under realistic operating conditions.

## Figures and Tables

**Table 1:** Average local elemental composition of raw FA.

Element	Content (wt%, Mean ± SD)
C	26.33 ± 9.85
O	25.51 ± 8.44
Ca	19.53 ± 5.39
Cl	16.64 ± 2.21
Na	6.37 ± 3.42
S	2.66 ± 0.24
K	1.62 ± 1.83
Pb	0.94 ± 2.98
Si	0.40 ± 0.45

**Table 2:** Particle size characteristics of raw FA and FA dispersed in DESs.

DES	FA Concentration (wt%)	*Dv*(10) (µm)	*Dv*(50) (µm)	*Dv*(90) (µm)	*D*[4,3] (µm)	Span (–)
–	Raw FA	2.86	21.3	82.6	36.0	3.74
ChCl/EG (1:10)	2%	1.42	9.25	22.4	12.0	2.27
	5%	2.26	12.9	33.5	16.7	2.45
	10%	2.12	18.1	50.6	23.9	2.68
	15%	4.83	25.2	61.3	31.3	2.28
	20%	7.18	33.0	77.5	40.4	2.13
ChCl/EG (1:5)	2%	0.95	5.97	17.3	8.16	2.73
	5%	2.80	13.3	31.4	16.1	2.15
	10%	3.68	20.1	50.0	25.3	2.34
	15%	5.91	29.1	68.9	35.6	2.17
	20%	8.75	36.1	82.5	43.6	2.04

**Table 3:** Average viscosity (mPa·s) of ChCl/EG-based FA dispersions at 25 °C.

DES (Molar Ratio)	FA Loading (wt%)					
	0	2	5	10	15	20
ChCl/EG (1:10)	18.1	21.4	27.4	39.4	53.3	87.8
ChCl/EG (1:5)	21.3	26.1	30.8	43.4	62.9	89.9

## Data Availability

The original contributions presented in this study are included in the article and Supplementary Materials. Further inquiries can be directed to the corresponding author.

## Supplementary Materials

The following supporting information can be downloaded at: https://www.mdpi.com/article/10.3390/molecules31173125/s1, Figure S1. Backscattering (BS) profiles of the 2 wt% FA dispersion in ChCl/EG (1:10) during the 3 h observation period at 25 °C. Figure S2. Backscattering (BS) profiles of the 20 wt% FA dispersion in ChCl/EG (1:10) during the 3 h observation period at 25 °C. Figure S3. Backscattering (BS) profiles of the 2 wt% FA dispersion in ChCl/EG (1:5) during the 3 h observation period at 25 °C. Figure S4. Backscattering (BS) profiles of the 20 wt% FA dispersion in ChCl/EG (1:5) during the 3 h observation period at 25 °C.
