# Supplementary material for: Waste-to-Heat: Thermophysical and Rheological Properties of Fly Ash Dispersions in Deep Eutectic Solvents
Source: Molecules. 2026 Sep 7;31(17):3125. doi: 10.3390/molecules31173125 (PMC13567244; doi:10.3390/molecules31173125)
Supplement: Supplementary file 1 [file molecules-31-03125-s001.zip › molecules-4490087-supplementary.pdf]

SUPPLEMENTARY MATERIALS

for

Waste-to-Heat: Thermophysical and Rheological Properties of Fly Ash Dispersions in Deep Eutectic Solvents

Bertrand Jóźwiak[1,*] and Grzegorz Dzido[2]

[1] Department of Chemical and Molecular Engineering, Faculty of Process and Environmental Engineering, Lodz University of Technology, Wólczańska 213, 93-005 Łódź, Poland

[2] Department of Chemical Engineering and Process Design, Faculty of Chemistry, Silesian University of Technology, Strzody 7, 44-100 Gliwice, Poland

*Correspondence: bertrand.jozwiak@p.lodz.pl

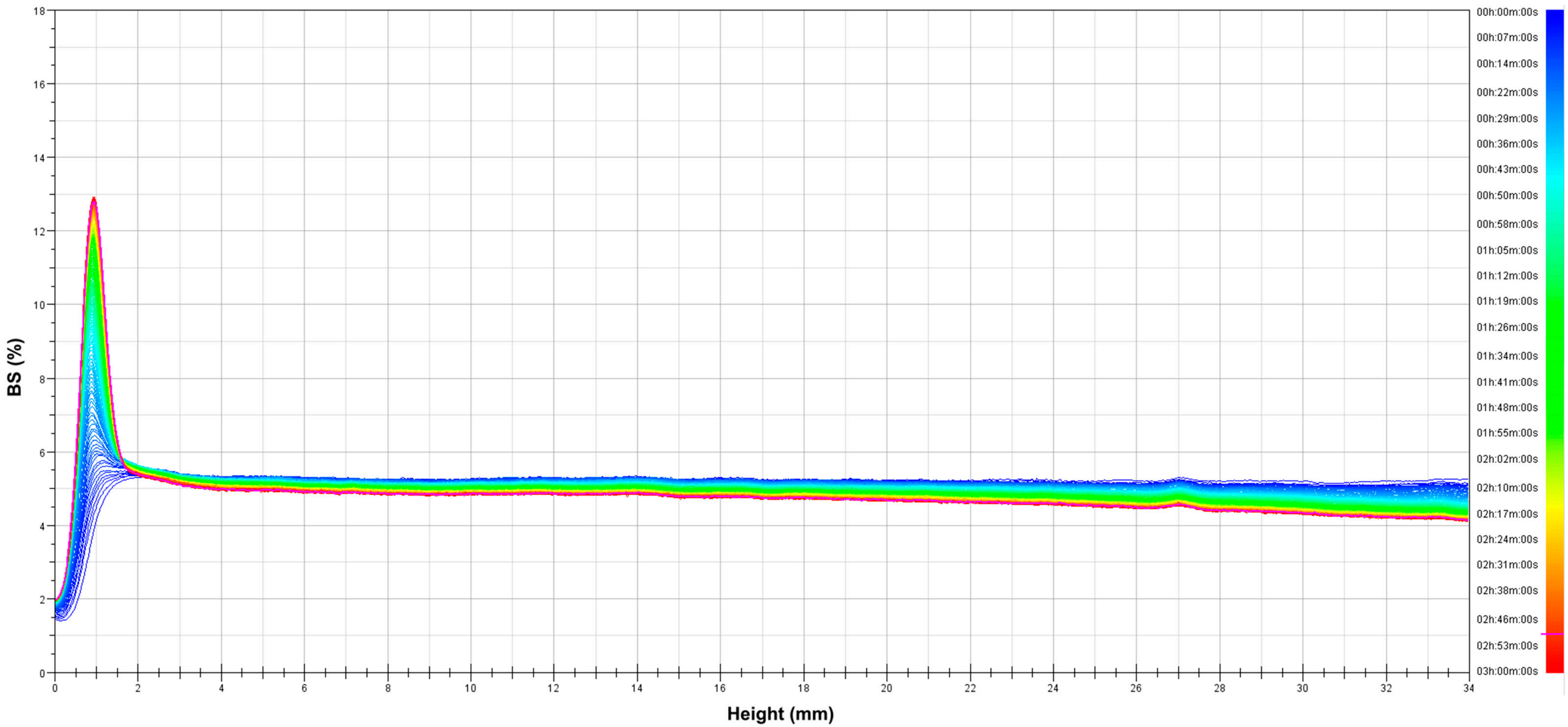

Figure S1. Backscattering (BS) profiles of the 2 wt% FA dispersion in ChCl/EG (1:10) during the 3 h observation period at 25 °C.

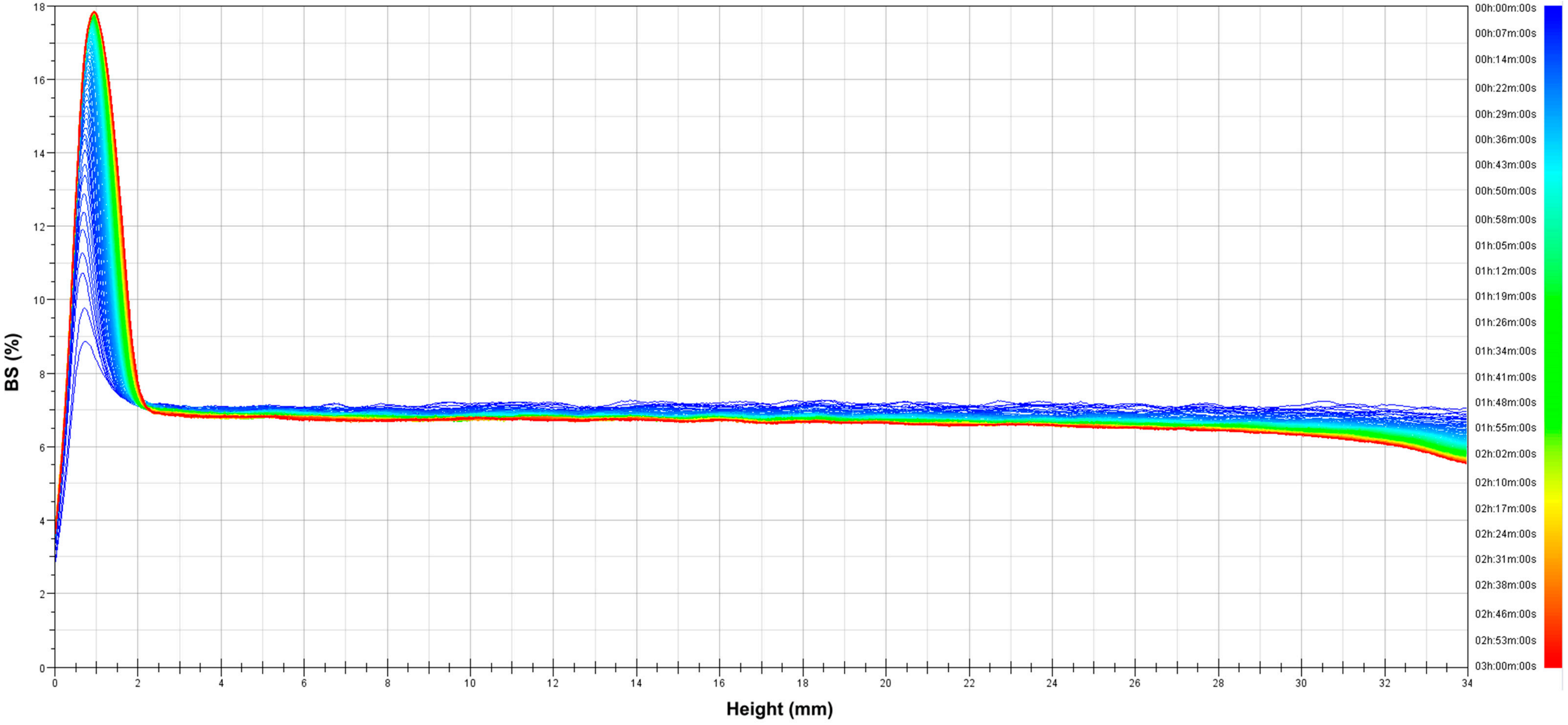

Figure S2. Backscattering (BS) profiles of the 20 wt% FA dispersion in ChCl/EG (1:10) during the 3 h observation period at 25 °C.

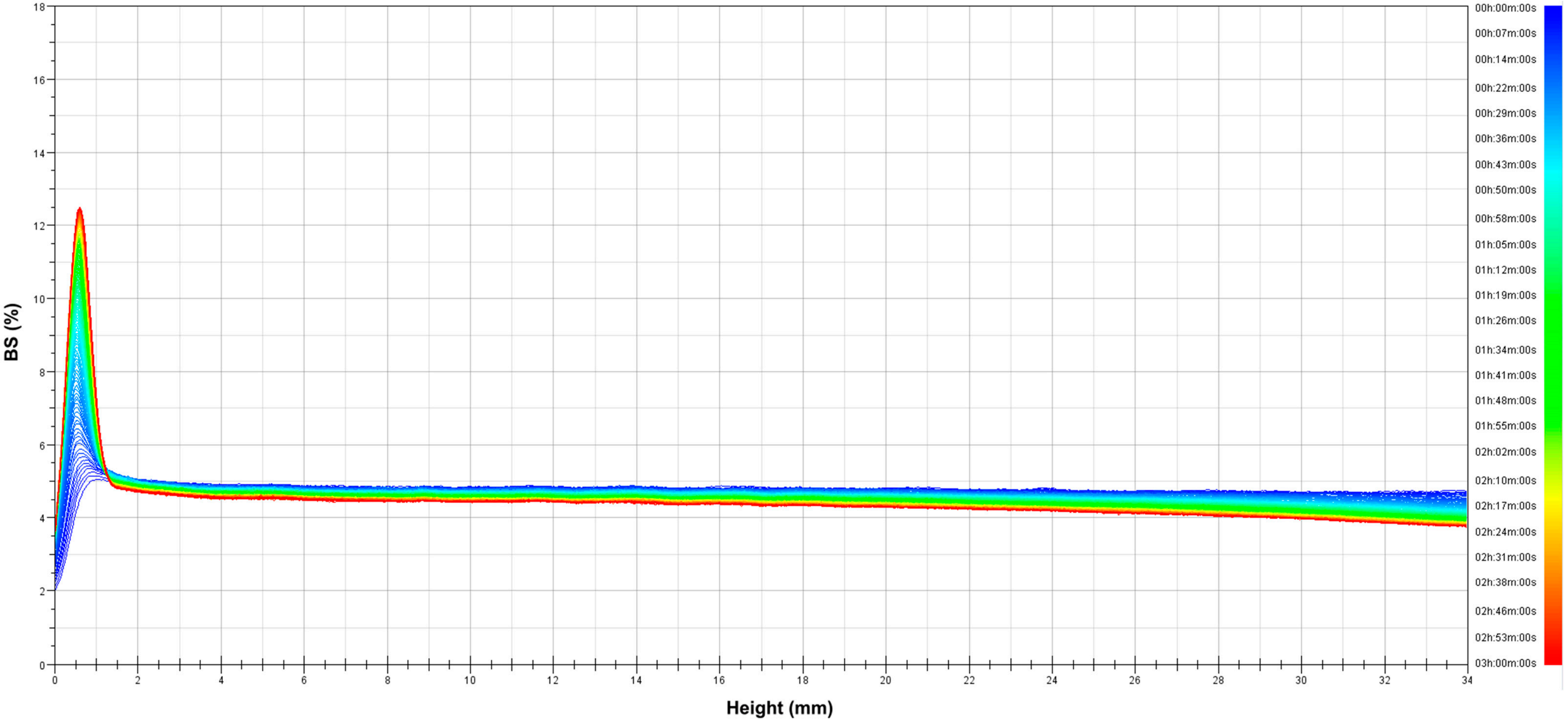

Figure S3. Backscattering (BS) profiles of the 2 wt% FA dispersion in ChCl/EG (1:5) during the 3 h observation period at 25 °C.

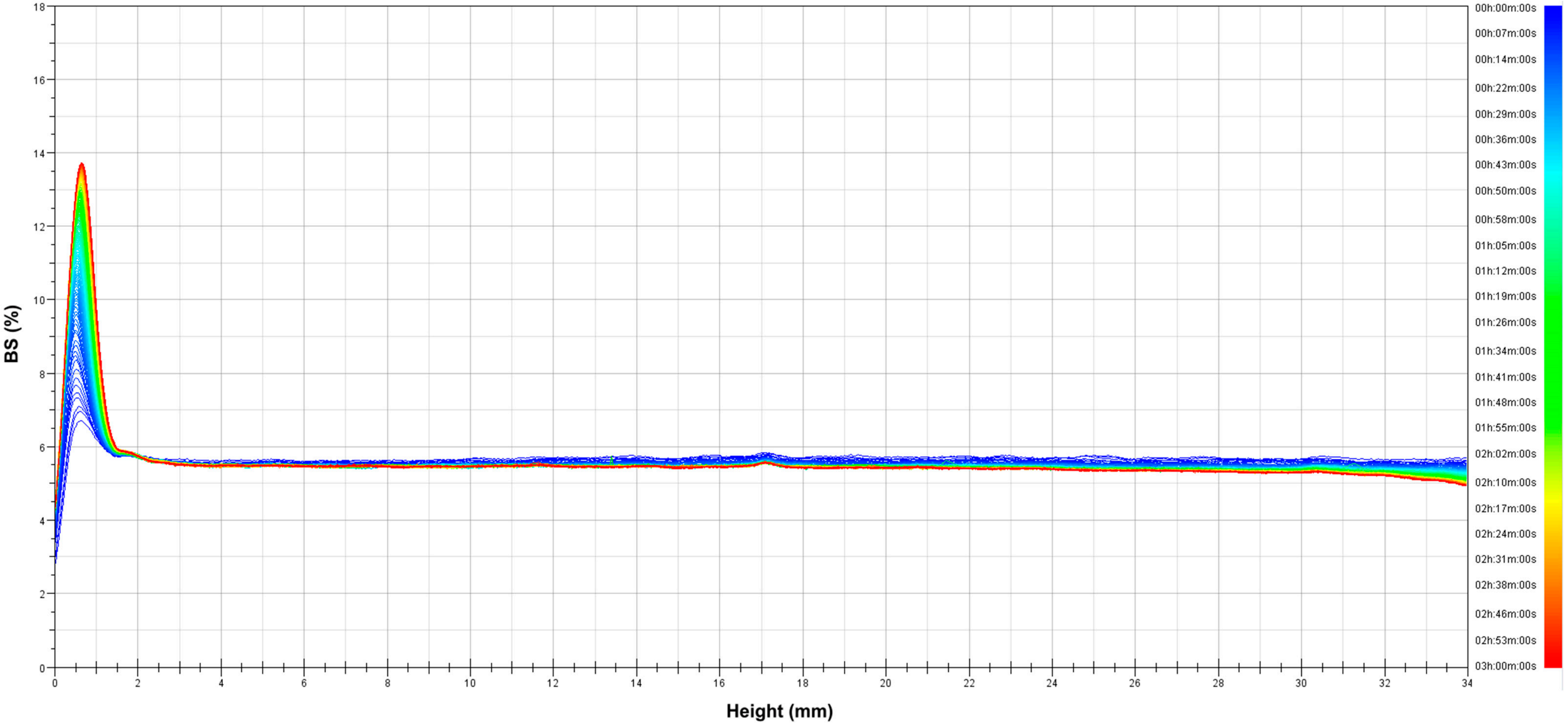

Figure S4. Backscattering (BS) profiles of the 20 wt% FA dispersion in ChCl/EG (1:5) during the 3 h observation period at 25 °C.
